# Study of muscle contraction induced by electrical pulse stimulation and nitric oxide in C2C12 myotube cells

**DOI:** 10.20463/jenb.2018.0004

**Published:** 2018-03-31

**Authors:** In Ho Lee, Yoon Jeong Lee, Hyobin Seo, Yi Seul Kim, Ju-Ock Nam, Byung-Duk Jeon, Tae-Dong Kwon

**Affiliations:** 1. Department of Leisure Sports, Kyungpook National University, Sangju Republic of Korea; 2. Department of Life Sciences, Yeungnam, University, Gyeongsan Republic of Korea; 3. Department of Food Science and Biotechnology, Kyungpook national University, Daegu Republic of Korea; 4. Institute of Agricultural Science & Technology, Kyungpook National University, Daegu Republic of Korea; 5. Departmentof Physical Education Leisure, Suseong College, Daegu Republic of Korea

**Keywords:** C2C12, Myotube cell, Electrical pulse stimulation, Nitric oxide, Skeletal muscle

## Abstract

**[Purpose]:**

This study aimed to examine the independent effect of electrical pulse stimulation(EPS) and nitric oxide(NO) on muscle contraction and their synergistic or combined effect on contraction phenomenon using C2C12 mouse skeletal muscle cells.

**[Methods]:**

Some differentiated C2C12 myotube cells were untreated (control). Other cells did not receive EPS and did receive 0.5, 1.0, or 2.0 mM of the NO donor, S-nitroso-N-acetyl-penicillamine (SNAP; -E/S0.5, -E/S1.0, and -E/S2.0, respectively). For the EPS treatments (0.3 V/mm, 1.0 Hz, and 4.0 ms), differentiated C2C12 myotube cells received only EPS or both EPS and the SNAPtreatments at the same concentrations (+E/-S, +E/S0.5, +E/S1.0, and +E/S2.0, respectively). All samples were then cultured for 4 days.

**[Results]:**

Differentiated C2C12 cellswere stimulated by the EPS, NO, and EPS+NO treatments. The cell length of the +E/S2.0 Group after the 4-day culture (84.2±13.2㎛) was the shortest of all the groups. The expressions of AMPK, JNK, Akt, eNOS, GLUT4, and PGC1α proteins were noticeably dominant. The results indicated synergistic effect on muscle contraction of simultaneously applied EPS and SNAP.

**[Conclusion]:**

Motor skills were significantly improved when exercise was accompanied by the intake of NO precursor and/or NO, compared to that upon their independent application or treatment.

## INTRODUCTION

Physical activity is crucial for health^1^. For athletes, physical activity is associated with a notable adaptation of skeletal muscle cells^1-4^. Elite athletes routinely incorporate ergogenic aids into their training to enhance their sports performance. Improving athletic performance is the focus of active ongoing research^5,6^. Studies of the basic cellular mechanisms behind the diverse reactions in the human body that are associated with the simultaneous use of ergogenic aids and physical activity have included animal models. These in vivo studies have faced various challenges.

A recent study hypothesized that a form of artificial electrical stimulation termed electrical pulse stimulation (EPS) can lead to the same reactions normally produced in vivoin skeletal muscle tissues in vitro^7^. To explore this hypothesis, the authors constructed a model of physiological electrical stimulus by determining EPS parameters including voltage, stimulus interval, and frequency. The results demonstrated that the system was appropriate to study intracellular metabolism upon acute muscle contraction^7^.

Two metabolic characteristics of skeletal muscles in response to physical activity are the increases in the number of mitochondria^8-10^ and oxidative capacity^4,10,11^. These characteristics are reported to occur in skeletal muscle cells stimulated by EPS. Among the ergogenic aids, the essential amino acid L-arginine enhances athletic performance by increasing the synthesis of creatine and nitric oxide (NO), which rapidly promotemaximum muscular strength^13-15^. NO is synthesized under the control of NO synthase (NOS)^16^. There are three unique isomers of NOS expressed by three different genes. Neuronal NOS (nNOS, NOS1) is found throughout the body. Inducible NOS (iNOS, NOS2) is produced by macrophages and regulated by cytokines. Endothelial NOS (eNOS, NOS3) is found in vascular endothelial cells and participates in vascular smooth muscle relaxation^17-19^. The expression of nNOS in skeletal muscles has been reported to increase during contusion^20^, muscle activation^21^, and aging^22^, while it decreases during denervation^23^. The expression of eNOS in skeletal muscles, in particular, has been reported to increase upon long-term exercise^21,24^. The expression of iNOS increases in individuals with diseases, such as chronic heart failure^25,26^ and autoimmune myopathy^27^, and in vitrowhen cultured skeletal muscle cells are exposed to bacterial endotoxin or inflammatory cytokines^28,29^. However, iNOS precursor is absent or present in trace amounts in healthy skeletal muscles of rats or mice^30-32^. The NO produced by these NOS isoforms has been associated with the generation of muscular strength^33^, blood flow control^34^, myoblast differentiation^35^, respiration^36^, and glucose homeostasis^37^ in skeletal muscles. Thus, as elucidated by numerous prior studies, EPS plays the same role as the nerve impulse that enhances muscle adaptation, and NO acts as the regulator in the increase of muscular strength in skeletal muscles.

This knowledge suggests that simultaneous exercise and intake of NO supplement are likely to synergistically influence athletic performance in terms of muscle adaptation and muscular strength. The present study aimed to provide the basic biochemical data of this scenario.

## METHODS

### Cells and treatment

#### C2C12 cell proliferation and differentiation

C2C12 mouse skeletal myoblasts purchased from the American Type Culture Collection (ATCC, Manassas, VA, USA) were dispensed in aliquots of 3×104 cellsto the wells of a 6-well plate. The cells were cultured in Dulbecco's Modified Eagle's Medium (DMEM) containing 2mM L-glutamine, 1% penicillin-streptomycin, and 10% fetal bovine serum (FBS) at 37℃ in an atmosphere of 5% CO2and adequate humidity. Once cell proliferation was suitable, the medium was switched to the differentiation medium (DMEM containing 1% non-essential amino acids, 1% penicillin-streptomycin, and 2% calf serum). Cells were allowed to differentiate during 4days of culture prior to the experiments. The C2C12 cells were treated in eight different ways (Table 1)

**Table 1.** Treatment groups

Groups	- EPS	Groups	+ EPS
CON	SNAP 0mM	+E/-S	SNAP 0mM
-E.S0.5	SNAP 0.5mM	+E/S0.5	SNAP 0.5mM
-E.S1.0	SNAP 1.0mM	+E/S1.0	SNAP 1.0mM
-E.S2.0	SNAP 2.0mM	+E/S2.0	SNAP 2.0mM

#### EPS treatment of C2C12 cells

The 6-well plate containing the differentiated C2C12 myotube cells was connected to the 6-well C-dish (Ion Optix Corp., Milton, MA, USA), which functioned as the electrical stimulation apparatus.A C-Pace EP electrical pulse generatorwas used to generate electrical pulses (0.3V/mm, 1.0Hz, 4.0ms) during a 4-day culture period.

#### NO treatment of C2C12 cells

The differentiated C2C12 myotubes were cultured for 4 days after being treated in the absence or presence (0.5mM, 1.0mM, 2.0mM) ofthe NO donor S-nitroso-N –acetylpenicillamine(SNAP, Santa Cruz Biotechnology, Dallas, TX, USA).

#### Simultaneous EPS-NO treatment of C2C12 cells

The differentiated C2C12 myotubes were simultaneously treated as described above for 4 days with the electrical pulses and SNAP.

### Analyses

#### Cell morphology and length

To investigate the effects of EPS and NO on muscle contraction, the morphology changes of differentiated C2C12 cells and the changes in cellular length as an indication of the degree of contraction were monitored and measured. Using the camera attached to the phase-contrast microscope, five randomly selected areas of each well for experimental group were photographed and the stored JPG files were analyzed using Image J software (NIH, Bethesda, MD, USA). For each area examined, the length of five muscle cells was measured using a 50-㎛ scale bar.

#### Quantification of NO produced and secreted by C2C12 cells

Quantification was carried out to estimate the amount of NO produced and secreted by C2C12 cells in each experimental group. NO was quantified in 100 ㎕ of culture filtrate by measuring the amount of nitrite ions (NO2-)produced as a reaction product of NO. The same volume of Griess agent (1% sulfanilamide, 0.1% naphthylethylenediamine dihydrochloride, 2.5% phosphoric acid) was added to the filtrate and left to react for 10 min at room temperature. The optical density was measured at 570 nm using a model 550 ELISA microplate reader(Bio-Rad, Hercules, CA, USA).A standard curve for the concentration of NO2- was constructed using NaNO3. The concentration of the produced NO was expressed in μM.

#### Western blot analysis of C2C12 cell proteins

Western blotting was carried out to investigate the level of expression of various proteins in C2C12 cells under the influence of EPS and NO. The treated cells were washed with cold phosphate buffered saline(PBS). The cells were collected using a scraper and suspended in lysis buffer(50mM Tris-HCl, pH 8.0, 5mM EDTA, 150mM NaCl, 0.5% Nonidet P-40, 1mM phenylmethylsulfonyl fluoride, 1㎍/ml aprotinin, 1㎍/ml pepstatin, and 1㎍/ml leupeptin in a volume of 100㎕). Protein from the lysed cells was recovered by centrifugation for 5 min at 4℃ and 2,000g. The protein concentration was measured using a NanoDrop spectrophotometer (Thermo Fisher Scientific, Waltham, MA, USA) and the proteins were denatured by heating for 5 min. The various proteins were resolved by SDS-PAGEand transferred toa nitrocellulose membrane. The membrane was incubated ina blocking solution consisting of 5% skim milk, 25mM Tris-HCl, 150 mM NaCl, and 0.2% Tween-20 for 30 min initially, then overnight with the suggested concentrations of antibodies to AMP-activated protein kinase (AMPK), c-Jun N-terminal kinase (JNK), protein kinase B (Akt), nNOS, eNOS, insulin-regulated glucose transporter (GLUT4), and peroxisome proliferator-activated receptor gamma coactivator 1-alpha(PGC1α). The membrane was washed three times for 10 min each time in TTBS. Following the final wash, the membrane was incubated with secondary antibody conjugated to horseradish peroxidase for one hour before washing as previously described. ECL Western Blotting Detection Reagents(Amersham, Buckinghamshire, UK) were used for the reaction, and the results were analyzed using an Image station 4000MM Pro Imaging System(Kodak, Rochester, NY, USA). For each experimental group, the concentration of proteins expressed relative to the standard(β-actin) was calculated and expressed as a percentage3.

### Statistical analyses

The statistical analyses were done using the SPSS/PC+18.0statistics program for Windows (SPSS Inc., Chicago, IL, USA). The results are expressed as mean ± standard error. To test the significance of the morphology changes in C2C12 cells on days 1 and 4, Student’s t-test was performed. To test the significance of morphology changes and NO concentration changes in C2C12 cells, one-way ANOVA was performed. Least Significant Difference (LSD) was used as post-hoc test. The level of significance was set atp< 0.05.

## RESULTS

### Morphological changes in differentiated C2C12 cells

**Fig.1.**
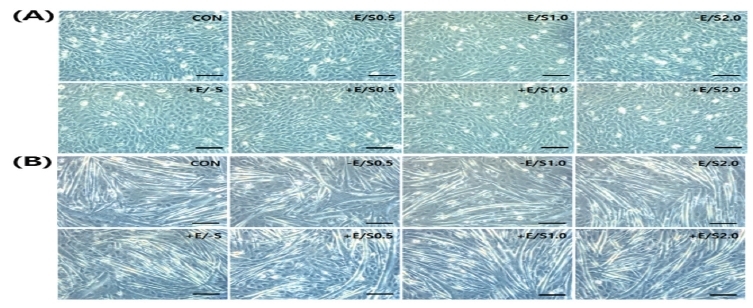
Cell morphology during C2C12 cell dfiefrentiation on day 1 (A) and day 4 (B)

The changes in C2C12 cell morphology with differentiation following the EPS treatment are presented in Fig. 1. When the cells were observed on days 1 and 4 during the cultivation in the differentiation medium, an overall increase in cellular length was evident, rather than changes in morphology due to EPS.

### Changes in length of C2C12 cells

**Fig.2.**
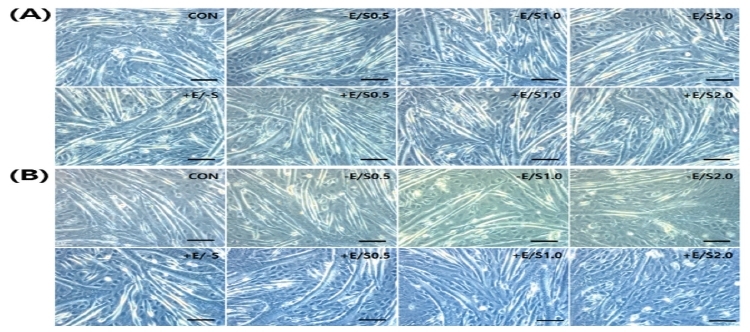
Morphology of C2C12 cells during dfiefrentiation at day 1 (A) and day 4 (B) in the presence of SNA wPith or without EPS

**Fig.3.**
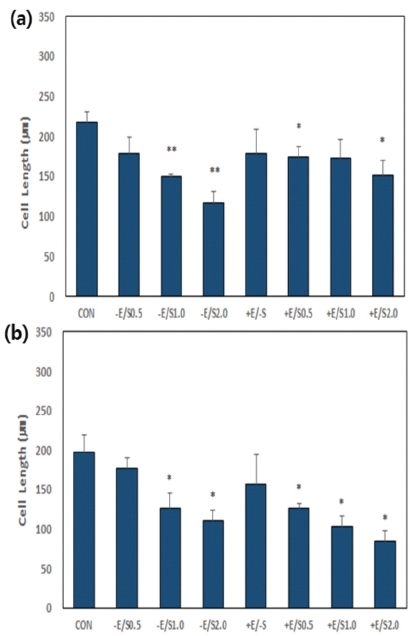
Measurement of cell length of the samples shown in Fig. 2

When cell length was measured on day 1 after the cells were treated in the culture medium, the lengths in all groups was shorter than that in the CON group. In particular, significantly shorter lengths were obtained from the –E/S1.0 group (*p*<0.01), –E/S2.0 group (*p*<0.01), +E/S0.5 group (*p*<0.05), and +E/S2.0 group (*p*<0.05) compared to those from the CON group. In all groups, cells became shorter as the concentration of SNAP increased. When cellular length on day 4 was measured, the lengths in all groups were shorter than that in the CON group. In particular, significantly shorter lengths were obtained from the –E/S1.0 group (*p*<0.05), -E/S2.0 group (*p*<0.05), +E/S0.5 group (*p*<0.05), +E/S1.0 group (*p*<0.05), and +E/S2.0 group (*p*<0.05) compared to those from the CON group. Comparison of sample pairs treated with the same concentration of SNAP in the absence or presence of EPS (i.e., -E/+S and +E/+S, respectively) revealed a shorter cell length with EPS, with progressive shortening as the SNAP concentration increased. Morphological changes with time of treatment are summarized in Figs. 2 and 3, and Table 2.

**Table 2.** Measurements of cell length

group	1day	4day
CON	218.1±12.5	196.7±22.4
-E.S0.5	179.0±19.5	177.5±12.1
-E.S1.0	149.5±3.7	126.5±19.3
-E.S2.0	117.2±13.2	110.4±13.2
+E/-S	179.2±30.1	156.8±38.1
+E/S0.5	174.7±13.0	125.9±6.8*
+E/S1.0	173.3±22.9	103.5±12.7*
+E/S2.0	151.5±18.0	84.2±13.2*

The data are from day 1 (a) and day 4 (b) in the presence of SNAP with or without EPS. *p<0.05 and **p<0.01 significantly different from CON.

### Changes in concentration of NO produced and secreted by C2C12 cells after each treatment

**Fig.4.**
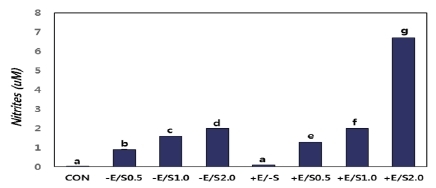
Measurement of nitric oxide content in the control and treatment groups.

Results of the NO produced and secreted from C2C12 cells after day 4 of stimulation in each experimental group are summarized in Fig. 4. A higher concentration of NO was observed in the +E/-S group versus the CON group, in +E/S0.5 versus -E/S0.5, in +E/S1.0 versus -E/S1.0, and in +E/S2.0 versus -E/S2.0. The comparisons of the groups not treated or treated with EPS revealed increasing NO levels as the concentration of SNAP increased. The +E/S2.0 group displayed a significantly higher increase in NO concentration compared to all other groups.

### Changes in protein expression in C2C12 cells after each treatment

**Fig.5.**
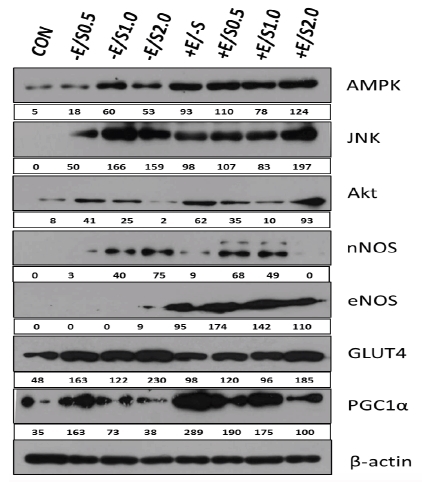
Protein expressioninC2C12 cells. β-actin was used as a control. The numbers representprotein expression presented as the percentage compared to the expression of β-actin

The level of AMPK expression was markedly increased in the experimental group where the cells were simultaneously treated with EPS and SNAP. In particular, the +E/S2.0 group exhibited higher protein expression than the CON group. The level of JNK expression was not detected in the CON group but was increased in the SNAP treatment group and EPS+SNAP combined treatment group. Although the -E/S0.5 group displayed a lower expression level than the other groups, the level was higher than the CON group. Akt protein expression was increased in the SNAP treatment group and EPS+SNAP combined treatment group, compared to that in the CON group. Notably, higher levels of expression were observed in the +E/-S, +E/S0.5, and +E/S2.0 groups, with the latter displaying the highest level. The expression of nNOS was higher in the +E/-S group versus the CON group, in +E/S0.5 versus -E/S0.5, and in +E/S1.0 versus -E/S1.0. No nNOS expression was detected in the +E/S2.0 group. eNOS expression was only evident in groups treated with EPS. The highest eNOS level was observed in the +E/S0.5 group. The level of GLUT4 expression was higher in the +E/-S group versus the CON group, but the level was lower in +E/S0.5 versus -E/S0.5, +E/S1.0 versus -E/S1.0, and +E/S2.0 versus -E/S2.0. The largest increase in GLUT4 expression level was observed in the –E/S2.0 group among the –E/+S groups and in the +E/S2.0 group among the – E/+S groups. The level of PGC1α expression was higher in the +E/-S group versus the CON group, +E/S0.5 versus -E/S0.5,+E/S1.0 versus -E/S1.0, and +E/S2.0 versus -E/S2.0. The largest increase in PGC1α expression level was observed in the +E/-S group. The results of protein expression levels are presented in Fig. 5.

## DISCUSSION

The loss of myofibril proteins in skeletal muscles caused by a disease or physical inactivity seriously diminishes muscular strength and function. In contrast, resistance training prevents the loss of myofibrils. Resistance training reportedly causes the differentiation and enlargement of muscles by stimulating their signaling^38^. This is because the electrical stimulation of somatic neurons leads to a notable adaptation of skeletal muscle cells^1-4^. Thus, the present study examined the effects of EPS and NO on muscle contraction, and whether the simultaneous treatment with EPS and NO enhanced muscle contraction in C2C12 mouse skeletal muscle cells.

When C2C12 cells were differentiated and treated with EPS for 4 days, the cellular length was shorter in the +E/-S group than in the CON group and in the +E/+S group than in the -E/+S group. The shortest cellular length was observed on day 4. The results indicate that EPS produced muscle contraction, which is consistent with a previous study that reported the shortened length of skeletal muscle cells as a consequence of EPS^39^. Presently, when the cells were treated with EPS, a higher level of NO secretion was observed in the +E/-S group than in the CON group, and in the +E/+S group than in the -E/+S group, suggesting that the electrical stimulation enhanced NO production. This agrees with the reports describing the increased nNOS expression in skeletal muscles upon muscular activity^21^ and increased NOS expression upon long-term exercise^21,24^. These present and prior results imply that the muscle contraction caused by EPS can increase NOS expression and the subsequent secretion of NO. The assessment of protein expression using western blotting revealed that the levels of AMPK, JNK, Akt, nNOS, eNOS, GLUT4, and PGC1α proteins were higher in the +E/-S group than in the CON group, while the expression of iNOS or ACC was not detected. This suggests that the muscle contraction caused by EPS increased NO secretion, which increases the activity of the GLUT4 sugar transporter and the consequent transport of glucose to skeletal muscles^40-43^. Simultaneously glycolysis is suppressed^44,45^ and the metabolic pathways in the muscle including mitochondrial respiration are regulated^46,47^, which facilitates the β-oxidation of fatty acids in the mitochondria and suppresses fatty acid synthesis.

When C2C12 cells were differentiated and treated with various concentrations of the NO donor SNAP for 4 days, the cellular length was shorter in the -E/+S group than in the CON group, and progressively shortened as the concentration of SNAP increased. The shortest cellular length was observed on day 4. These observations support the view that muscle contraction occurs due to NO. This is also supported by the description of the increased muscle contraction capacity when cells were provided with an external source of NO, which increased the movement of calcium ions in the sarcoplasmic reticulum^47^. In addition, studies have reported that NO production that occurs following the activation of NOS by insulin and muscle contraction regulates the influx of glucose into skeletal muscle and promotes improved athletic performance^21,48,49^. This implies that the NO provided by the SNAP treatment may have acted analogously as the NO produced by NOS that is activated by insulin or muscle contraction. When the cells were treated with SNAP, a higher level of NO secretion was observed in the –E/+S group than in the CON group, and progressively increased as the SNAP concentration increased, suggesting that SNAP led to enhanced NO secretion. SNAP is anNO donor. Hence, the increased concentration of SNAP may have yielded levels of NO. However, comparison of the levels of NO secretion in the -E/+S and +E/+S samples treated with identical SNAP concentration revealed a significantly higher level of NO in the +E/+S group than in the – E/+S group, especially in the +E/S2.0 group, indicating that the measured level of NO was not a result of the concentration of SNAP. Western blotting showed that the levels of AMPK, JNK, Akt, nNOS, GLUT4, PGC1α proteins were higher in the –E/+S group than in the CON group. This supports the suggestion that NO can cause muscle contraction, which leads to an increased production of nNOS expression increased, thereby increasing NO secretion. The resulting increased glucose influx into the muscle cells would facilitate the β-oxidation of fatty acids in the mitochondria.

Presently, when differentiated C2C12 cells were simultaneously treated with EPS and various concentrations of SNAP for 4 days, a markedly shorter cellular length was observed in the +E/+S group than in the CON group, and the length progressively decreased as the SNAP concentration increased. On day 4, the cellular length of the +E/S2.0 group was 84.2±13.2㎛, which was the shortest among all experimental groups. When the cells were simultaneously treated with EPS and SNAP, a higher level of NO secretion was observed in the +E/+S group than in the CON group as the SNAP treatment concentration increased, with the highest level of NO observed in the +E/S2.0 group. The results concerning cellular length and NO secretion supported the view that the simultaneous treatment of EPS and NO enhanced muscle contraction. Western blotting showed that the levels of AMPK, JNK, Akt, eNOS, GLUT4, and PGC1α proteins were higher in the +E/+S group than in the CON group, with a predominantly higher level observed in the +E/S2.0 group. These observations suggest that the simultaneous treatment with EPS and NO enhanced muscle contraction, increased the eNOS expression so that NO secretion increased, and increased the influx of glucose into the muscle cells to facilitate the β-oxidation of the fatty acids in the mitochondria. However, the expression of nNOS was markedly lower in the +E/S2.0 group, which is so far supported by a single report concerning the reduction of nNOS expression during denervation23. It is conceivable that EPS and the enhanced production of NO may have created an environment similar to that of denervation in C2C12 cells.

The present results demonstrate the synergistic action of EPS and NO in skeletal muscle cells. The data should prove valuable as basic information to investigate the biological phenomena occurring in individual organs. It is essential that future in vivo studies clinically verify whether simultaneous exercise and intake of NO precursor or NO supplement enhances athletic performance and whether the simultaneous treatment has a positive influence on the enhancement of athletic performance.

## CONCLUSION

This study suggests the possibility that simultaneous exercise and intake of NO precursor or NO supplement helps enhance athletic performance. Systematic studies are needed to reveal the detailed mechanisms of the signaling pathway and interaction between physical activity and NO intake. The ultimate outcome could be a treatment regimen that benefits the performance of elite athletes as well as the general public.
